# Supplementary material for: Endophytic Fungal and Bacterial Microbiota Shift in Rice and Barnyardgrass Grown under Co-Culture Condition
Source: Plants (Basel). 2022 Jun 16;11(12):1592. doi: 10.3390/plants11121592 (PMC9231121; doi:10.3390/plants11121592)
Supplement: Supplementary file 1 [file plants-11-01592-s001.zip › plants-1750207-supplementary.pdf]

Table S1. Bacterial community composition at genus level in soil

Genus	Soil 1	Soil 2	Soil 3	Soil 4	Soil 5	Soil 6
Bacillus	0.134176	0.116903	0.086166	0.156176	0.108563	0.097974
SC-I-84(f)	0.034790	0.04819	0.041810	0.044534	0.050242	0.043056
Nocardioides	0.022199	0.020587	0.020276	0.021427	0.069078	0.072689
MND1	0.032120	0.039929	0.039663	0.036208	0.027348	0.035585
67-14(f)	0.030474	0.029439	0.031288	0.032923	0.032155	0.036429
Gaiella	0.028517	0.026357	0.032546	0.027691	0.03247	0.032756
TK10(c)	0.038971	0.032061	0.032699	0.021618	0.007999	0.009835
Ellin6067	0.020598	0.022882	0.028896	0.022993	0.016708	0.019629
Lapillicoccus	0.006139	0.007212	0.005399	0.007142	0.063089	0.041072
Candidatus_Solibacter	0.015215	0.016981	0.021043	0.014743	0.015999	0.014141
MB-A2-108(c)	0.016550	0.017768	0.015951	0.016691	0.015486	0.017265
Pseudolabrys	0.014726	0.018227	0.018926	0.018753	0.013556	0.013508
Fictibacillus	0.016816	0.011474	0.008528	0.013368	0.021279	0.018236
Gaiellales(o)	0.012857	0.012261	0.014632	0.012719	0.016275	0.016294
Sphingomonas	0.013702	0.013310	0.014785	0.012910	0.011309	0.017096
Pedosphaeraceae(f)	0.012724	0.013572	0.012914	0.012680	0.012334	0.012073
Methyloligellaceae(f)	0.009609	0.009245	0.010767	0.010618	0.013556	0.012157
Gemmatimonadaceae(f)	0.012768	0.010490	0.011564	0.009510	0.009182	0.012748
TRA3-20(f)	0.010188	0.011998	0.013405	0.010121	0.007527	0.010595
Xanthobacteraceae(f)	0.008097	0.010425	0.010982	0.010198	0.012058	0.011650
Ramlibacter	0.011656	0.011474	0.010920	0.011611	0.006778	0.011524
Pseudomonas	0.015393	0.012130	0.014018	0.010847	0.001458	0.002068
Anaeromyxobacter	0.008586	0.009835	0.012423	0.010121	0.005596	0.007472
Nitrospira	0.009698	0.009900	0.010675	0.008823	0.006857	0.007345
mle1-7	0.007830	0.008130	0.010123	0.008326	0.007054	0.007260
Geobacteraceae(f)	0.008408	0.009441	0.011503	0.008632	0.003074	0.006796
S085(o)	0.009787	0.011736	0.011472	0.006187	0.002837	0.004812
Terrabacter	0.001913	0.002754	0.002577	0.002521	0.017417	0.013592
Pseudarthrobacter	0.000712	0.000262	0.000552	0.000573	0.024983	0.006965
Arthrobacter	0.000489	0.000852	0.000828	0.000649	0.016314	0.007260
others	0.434291	0.434173	0.442669	0.418685	0.351421	0.380118

Table S2. Fungal community composition at genus level in soil

OTU ID	Soil 1	Soil 2	Soil 3	Soil 4	Soil 5	Soil 6
Coprinellus	0.230134	0.114441	0.346799	0.134601	0.252542	0.456027
Sordariales(o)	0.092263	0.125837	0.059661	0.049287	0.195820	0.155501
Emericellopsis	0.026573	0.091599	0.033906	0.296464	0.035285	0.029920
Preussia	0.220604	0.024477	0.097961	0.059073	0.037355	0.056850
Sordariomycetes(c)	0.053733	0.048010	0.028770	0.094869	0.028744	0.033676
Penicillium	0.029818	0.126629	0.036435	0.030993	0.028923	0.013542
Fungi(k)	0.037968	0.077316	0.045455	0.025193	0.035643	0.018090
Apiosordaria	0.057770	0.005979	0.026573	0.010297	0.033625	0.100746
Branch03(o)	0.021079	0.141142	0.013593	0.019393	0.029792	0.009607
Aspergillus	0.040600	0.029766	0.031836	0.052072	0.032245	0.023277
Talaromyces	0.028821	0.017093	0.027901	0.032475	0.037176	0.010808
Chaetomium	0.035107	0.015739	0.018141	0.032781	0.037406	0.010987
Zopfiella	0.008406	0.014947	0.018805	0.008994	0.053605	0.010859
Thielavia	0.005468	0.007716	0.028974	0.015688	0.006924	0.007614
Acrophialophora	0.008406	0.009709	0.015100	0.012060	0.008483	0.002708
Fusarium	0.004855	0.004650	0.018652	0.010706	0.007512	0.004037
Neocosmospora	0.010092	0.003475	0.010271	0.005570	0.009454	0.004625
Alphamycetaceae(f)	0.004574	0.004395	0.029255	0.001226	0.001840	0.000945
Rozellomycota(p)	0.004165	0.013440	0.008023	0.006285	0.006950	0.003347
Curvularia	0.003756	0.004727	0.006260	0.006541	0.012418	0.002759
Scedosporium	0.004344	0.013210	0.001814	0.002095	0.002759	0.001175
Acremonium	0.000894	0.001022	0.000460	0.016403	0.002530	0.000818
Cercophora	0.000792	0.000869	0.000537	0.001686	0.013542	2.56E-05
others	0.069702	0.071593	0.094818	0.075247	0.076039	0.042056

Table S3. Fungi showed pathogenicity in barnyardgrass at genus level

Growth stages	genus
BBCH 17	*Curvularia, Acrophialophora, Gibellulopsis, Clonostachys, Ramichloridium, Ramichloridium, Podosphaera, Hawksworthiomyces, Hortaea, Gibellulopsis, Lectera, Curvularia*
BBCH 24	*Curvularia, Acrophialophora, Gibellulopsis, Clonostachys, Ramichloridium, Ramichloridium, Podosphaera, Hawksworthiomyces, Hortaea, Gibellulopsis, Lectera, Curvularia*
BBCH 37	*Curvularia, Gibellulopsis, Gibberella, Gibberella, Ramularia, Strelitziana, Exserohilum, Peroneutypa, Phaeophleospora, Ramularia*
BBCH 45	*Peroneutypa, Volutella, Veronaea, Exserohilum, Gibberella, Stagonospora, Moesziomyces, Cylindrocarpon*
BBCH 57	*Curvularia, Acrophialophora, Gibellulopsis, Gibberella, Ramularia, Clonostachys, Strelitziana, Pseudocercospora, Ramichloridium, Exserohilum, Stagonosporopsis, Erysiphe, Pestalotiopsis, Stagonospora, Passalora, Phlyctochytrium, Dirkmeia*

Table S4. The vital bacteria in rice groups

Node_Name	Degree Centrality	Closeness Centrality	Betweenness Centrality
Schlegelella	0.108696	0.386555	0.001612
Phenylobacterium	0.173913	0.429907	0.004738
Bradyrhizobium	0.173913	0.429907	0.004738
Haliangium	0.173913	0.429907	0.005048
Bacteroidales(o)	0.195652	0.438095	0.006291
Ideonella	0.195652	0.438095	0.006291
Rhizobiaceae(f)	0.043478	0.330935	0.008191
Rhodococcus	0.217391	0.446602	0.008332
Ralstonia	0.217391	0.446602	0.008332
Comamonadaceae(f)	0.217391	0.446602	0.008332
Sideroxydans	0.217391	0.446602	0.008332
Stenotrophomonas	0.217391	0.446602	0.008332
Xanthomonadaceae(f)	0.217391	0.446602	0.008332
Burkholderia-Caballeronia-Paraburkholderia	0.043478	0.321678	0.010030
Pleomorphomonadaceae(f)	0.217391	0.455446	0.016766
Rhodocyclaceae(f)	0.217391	0.455446	0.016766
Rhodocyclaceae(f)	0.217391	0.455446	0.016766
Delftia	0.239130	0.464646	0.020241
Pseudomonas	0.239130	0.464646	0.020241
Gallionella	0.195652	0.429907	0.028086
Gallionellaceae(f)	0.239130	0.474227	0.047516
Chthonobacter	0.065217	0.422018	0.048066
possible_genus_04	0.195652	0.505495	0.218963

Table S5. The vital fungi in rice groups

Node Name	Degree Centrality	Closeness Centrality	Betweenness Centrality
Sordariales(o)	0.108696	0.389831	0.071670
Cladosporium	0.152174	0.396552	0.042995
Acrophialophora	0.217391	0.418182	0.267963
Vishniacozyma	0.282609	0.460000	0.028120
Pleosporaceae(f)	0.347826	0.425926	0.017526
Tetracladium	0.347826	0.425926	0.022430
Alternaria	0.413043	0.522727	0.047928
Solicoccozyma	0.413043	0.522727	0.047928
Tausonia	0.413043	0.522727	0.047928
Fusarium	0.434783	0.534884	0.056858
Glomerellales(o)	0.434783	0.534884	0.147014
Filobasidium	0.456522	0.547619	0.077631
Thermomyces	0.478261	0.560976	0.121110

Table S6. The vital bacteria in barnyardgrass groups

Node Name	Degree Centrality	Closeness Centrality	Betweenness Centrality
Geobacter	0.032258	0.302439	0.000121
Rhizobiales_Incertae_Sedis(f)	0.032258	0.291080	0.000193
Treponema	0.032258	0.314721	0.000737
Dechloromonas	0.080645	0.311558	0.001082
Micromonosporaceae(f)	0.064516	0.321244	0.001455
Hungateiclostridiaceae(f)	0.080645	0.324607	0.001724
Pseudactinotalea	0.096774	0.328042	0.003042
Ralstonia	0.064516	0.314721	0.003141
Clostridium_sensu_stricto_1	0.129032	0.335135	0.006212
Fibrobacteraceae(f)	0.032258	0.358382	0.006872
Paludibacteraceae(f)	0.048387	0.400000	0.013035
Massilia	0.145161	0.380368	0.018088
Methylophilaceae(f)	0.177419	0.366864	0.020010
Streptomyces	0.048387	0.405229	0.023338
Gallionella	0.096774	0.416107	0.032187
Sideroxydans	0.096774	0.416107	0.032187
Gallionellaceae(f)	0.096774	0.416107	0.032187
Rhodocyclaceae(f)	0.048387	0.394904	0.044992
Burkholderia-Caballeronia-Paraburkholderia	0.225806	0.405229	0.047708
Rhodococcus	0.306452	0.433566	0.089451
Bacillus	0.306452	0.433566	0.089451
Methylocystis	0.177419	0.459259	0.116417
Pseudomonas	0.161290	0.452555	0.150900

Table S7. The vital fungi in barnyardgrass groups

Node Name	Degree Centrality	Closeness Centrality	Betweenness Centrality
Scolecobasidium	0.048387	0.378049	0.001084
Exserohilum	0.032258	0.316327	0.002862
Phaeosphaeriopsis	0.080645	0.387500	0.004240
Helotiales(o)	0.080645	0.387500	0.005239
Ascobolus	0.064516	0.397436	0.006515
Tremellaceae(f)	0.080645	0.387500	0.006737
Thielavia	0.064516	0.397436	0.014272
Albifimbria	0.112903	0.413333	0.016282
Aspergillus	0.145161	0.407895	0.019798
Fungi(k)	0.145161	0.407895	0.019798
Didymellaceae(f)	0.096774	0.392405	0.026070
Ascomycota(p)	0.161290	0.413333	0.029296
Glomerellales(o)	0.161290	0.430556	0.033501
Chytridiomycota(p)	0.161290	0.430556	0.033501
Penicillium	0.161290	0.413333	0.034316
Phoma	0.161290	0.413333	0.037049
Epicoccum	0.161290	0.430556	0.038152
Acremonium	0.177419	0.418919	0.043604
Pseudopithomyces	0.129032	0.402597	0.063354
Apodus	0.193548	0.424658	0.068320
Acrophialophora	0.403226	0.543860	0.509230

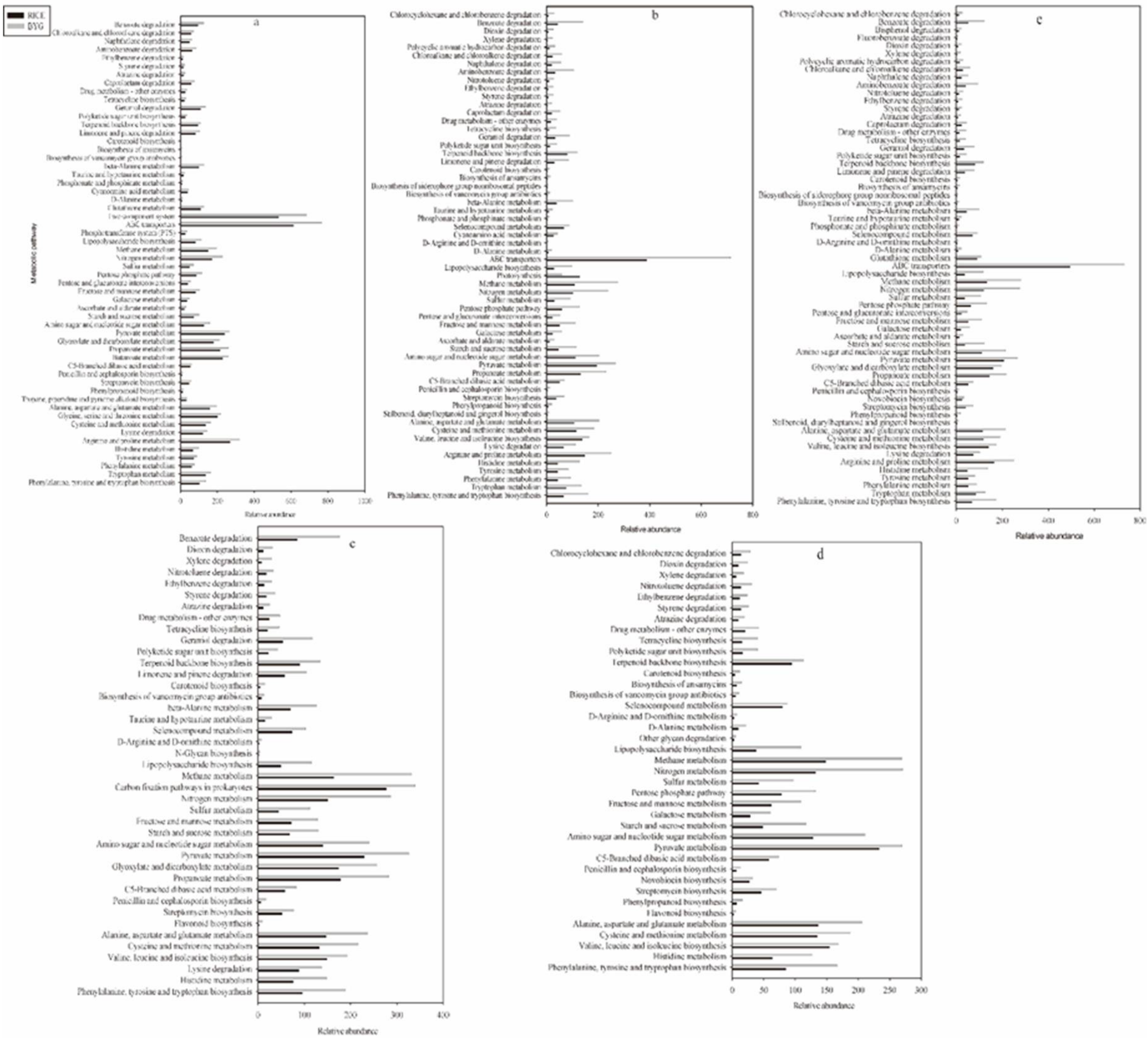

Figure S1. The significant microbial functional features in barnyardgrass at different growth stages.
